# 

**Published:** 1878-08

**Authors:** 


### CONTENTS.

ORIGINAL.

ART. I.—Vital Force. Address by Dr. A. R. Shank, 1

ART. II.—Surgical Cases in the private practice of Prof. Julius F. Miner. Reported by Charles A. Wall, B. S., 10

ART. III.—Glycerite of Animal Hypophosphites. By Elwood C. Lester, M. D., 13

Correspondence, 17

MISCELLANEOUS.

The Metric System in a Nut Shell, 19

Suits for Malpractice in Maine 21

Employment of Alcohol in Health and in Disease, 22

Occasional Non-Significance of Albuminuria, 24

Death under Ether, 24

Removal of a Portion of a Catheter from the Bladder, 25

Treatment of Pleurisy and Empyema, 26

Physiological and Therapeutic properties of Mercury, 28

On Telegraphists' Cramp 29

The Decimal System of Notation, 30

On Pruritus Vulvæ and Diabetes, 31

Science in Vanderbilt University, 32

EDITORIAL.

Buffalo Medical and Surgical Journal, Vol. xviii. 33

Popular Science Monthly, 34

Items in Brief, 35

Rural Hygiene, 36

Counter Prescribing 37

BOOKS REVIEWED:

Fifth Annual Report of the State Commissioner in Lunacy 37

Physics of the Infectious Diseases. E. A. Logan, 38

Congenital Occlusion and Dilation of Lymph Channels. Samuel C. Busey, 38

Atlas of the Diseases of the Skin. Balmanno Squire, 39

The Obstetric Gazette, 39

The Cincinnati Lancet and Observer, 39

Books and Pamphlets Received, 39

